# Diversity of sex chromosome abnormalities in a cohort of 95 Indonesian patients with monosomy X

**DOI:** 10.1186/1755-8166-4-23

**Published:** 2011-10-12

**Authors:** Nanis S Marzuki, Helena W Anggaratri, Lita P Suciati, Debby D Ambarwati, Chrysantine Paramayuda, Hannie Kartapradja, Aman B Pulungan, Alida Harahap

**Affiliations:** 1 Eijkman Institute for Molecular Biology, Jl. Diponegoro 69, Jakarta, 10430, Indonesia; 2 Endocrinology Division, Department of Child Health, Faculty of Medicine, University of Indonesia, Jl. Diponegoro 71, Jakarta, 10430, Indonesia

**Keywords:** sex chromosomes, monosomy X, karyotype, FISH

## Abstract

**Background:**

Monosomy × or 45,X is a cytogenetic characteristic for Turner syndrome. This chromosome anomaly is encountered in around 50% of cases, but wide variations of other anomalies have been found. This report is to describe the cytogenetic characteristics of 45,X individuals. To the best of our knowledge, there were no large series of 45,X cases has been reported from Indonesia.

**Results:**

Ninety five cases with 45,X cell line found, of which 60 were detected by karyotyping, 4 by FISH for sex chromosomes, and 31 by both karyotyping and FISH. Using karyotyping 37 out of 91 cases(40.6%) were identified as 45,X individuals, while cases who underwent FISH only 4 out of 35 cases (11.4%) showed 45,X result, resulting in total of 39 45,X cases (41.1%), and the rest 56 (58.9%) cases are mosaic. Among these cases, 21 out of 95 (22.1%) have Y or part of Y as the second or third sex chromosome in their additional cell lines. Result discrepancies revealed in 22 out of 31 cases who underwent both FISH and karyotyping, of which 7 showed normal 46,XX or 46,XY karyotypes, but by FISH, additional monosomy × cell line was found. Most of the cases were referred at the age of puberty (8-13 years old) or after that (14-18 years old), 31 and 21 cases respectively, and there were 14 cases were sent in adulthood.

**Conclusion:**

Wide variations of sex chromosome aberrations have been detected using the combination of conventional cytogenetic and FISH, including detection of low level of mosaicism and Y-chromosome fragments. Result discrepancies using both techniques were found in 22/31 cases, and in order to obtain a more details of sex chromosome constitution of individuals with 45,X cell line both FISH and karyotyping should be carried out simultaneously.

## Background

Monosomy × or 45,X is a cytogenetic characteristic for Turner syndrome (TS). This chromosome anomaly is encountered in around 50% of cases, but a wide variation of other anomalies of × chromosome have been found, including mosaicism, Xp or Xq deletion, dicentric × chromosomes, and isochromosomes of the × long arm [1-3].

Despite short stature, which seems to be the general clinical characteristic of TS, all other clinical stigmata are inconsistent, even in individuals with non-mosaic 45,X. Possible explanation for this fact is that the physical manifestations of TS patients largely depends on the karyotype [1], although parental origin of the × chromosome also can contribute to their phenotypes [4]. Patients with mosaic for 46,XX or iXq results in milder phenotype [1,3], while patients with mosaicism for 46,XY cell line or structural rearrangement of the Y chromosome mostly have masculinized external genitalia and are at increased risk for having gonadoblastoma and other gonadal tumors [1,5]. Furthermore, conventional cytogenetic method missed the Y component up to 9.3% [6]. This study describes the cytogenetic characteristics of 45,X individuals, who were referred to our clinic and not limited to female cases only. To the best of our knowledge, there were no large series of 45,X cases has been reported from Indonesia.

## Results

Ninety five cases with 45,X cell line found in our series, of which 60 were detected by karyotyping, 4 by FISH, and 31 by both karyotyping and FISH (Table 1). Cases, who underwent karyotyping technique, were 91. Seven of them resulted in normal sex chromosome karyotype, and 37 (40.6%) were 45,X individuals, while cases who underwent FISH method, only 4 out of 35 cases (11.4%) showed monosomy X, resulting in total of 39 45,X cases (41.1%), and the rest 56 (58.9%) cases are mosaic. Twenty one out of 95 cases (22.1%) have Y or Y segments as the second or third chromosome in their additional cell lines.

**Table 1:** Results of cytogenetic analysis in monosomy × patients

Karyotype and FISH results	Karyotyping only (Number of cases)	FISH only (Number of cases)	Karyotyping and FISH* (Number of cases)
45,X and/or nuc ish (DXZ1x1)	35	2	2

mos 45,X/46,XX and nuc ish (DXZ1x1)//(DXZ1x2)	4	0	1
nuc ish (DXZ1x1)//(DXZ1x2)//(DXZ1x3)	0	0	2
mos 45,X/47,XXX	1	0	0

mos 45,X/46,X,i(X)(q10)	10		
mos 45,X/46,X,idic(X)(q22)	1		
mos 45,X/46,X,i(X)(q10)/47,X,i(X)(q10)+Xp	1		
mos 45,X/46,X,i(X)(q10)/47,X+i(X)(q10)x2	2		

mos 45,X/46,XY and/or nuc ish (DXZ1x1)//(DYZ3x1)	1	1	4
mos 45,X/46,XY/46,X,+mar	1		
nuc ish (DXZ1x1)[256]//(DXZ1x2)[1]//(DXZ1,DYZ3)[40]//(DXZ1,DYZx2)[3]		1	

mos 45,X/46,X,r(Y)(p?q?)	1		
mos 45,X/46,X,del(Y)(q10)	1		
mos 45,X/46,X,inv(Y)	2		

Total	60	4	9

FISH: Fluorescence In Situ Hybridization; * data presented only for cases showed no results discrepancies in karyotyping and FISH.

The analyses were performed using karyotyping, FISH, and combination of karyotyping and FISH techniques. Sixty cases underwent karyotyping only, four cases FISH only. Nine cases, who underwent both karyotyping and FISH, showed no results deviations.

Cases that were analyzed by both karyotyping and FISH techniques, mostly showed different results (22 out of 31 cases). Seven out of 31 cases (22.5%), which found to be normal 46,XX or 46,XY by karyotyping, were revealed abnormal with additional monosomy × cell lines by FISH. On the other hand several mosaic monosomy × cases, which can be detected only by its proportion using FISH, can be showed its structural rearrangements using karyotyping technique (Table 2).

**Table 2:** Results discrepancies of karyotyping and FISH

No	Karyotyping	FISH	Number of cases
1.	46,XX	nuc ish (DXZ1x1)[≤ 30]//(DXZ1x2)[≥ 270]	5

2.	46,XX	nuc ish (DXZ1x1)[29]//(DXZ1x2)[266]//(DXZ1x3)[5]	1

3.	46,XY	nuc ish (DXZ1x1)[12]//(DXZ1,DYZ3)x1[288]	1

4.	mos 45,X[1]/46,X,del(X)(p10)[39]	nuc ish (DXZ1x1)[60]//(DXZ1x2)[240]	1

5.	mos 45,X[31]/46,X,i(X)(q10)	nuc ish (DXZ1x1)[240]//(DXZ1x2)[60]	1

6.	mos 45,X [7]/46,X,r(X)(p?q?)[9]	nuc ish (DXZ1x1)[167]//(DXZ1x2)[133]	1

7.	mos 45,X[9]/46,X,idic(X)(q23)[31]	nuc ish (DXZ1x3)[200]	1

8.	mos 45,X[4]/46,XY [26]	nuc ish (DXZ1x1)[18]//(DXZ1,DYZ3)x1[258]//(DXZ1x1,DYZ3x2)[24]*	1

9.	mos 45,X[28]/46,XY[12]	nuc ish (DXZ1x1)[224]//(DXZ1,DYZ3)x1[49]//(DXZ1x1,DYZ3x2)[27]*	1

10.	mos 45,X[30]/47,XY,+mar[10]	nuc ish (DXZ1x1)[252]//(DXZ1,DYZ3)x1[48]	1

11.	mos 45,X[20]/46,X,r(Y)(p?q?)[20]	nuc ish (DXZ1x1)[135]//(DXZ1,DYZ3)x1[165]	1

12.	mos 45,X[23]/46,X,+mar[17]	nuc ish (DXZ1x1)[178]//(DXZ1x2)[122]	1

13.	mos 45,X[25]/46,X,del(Y)(q11.23)[15]	nuc ish (DXZ1x1)[102]//(DXZ1,DYZ3)x1[198]	1

14.	mos 45,X[1]/46,X,idic(Y)(p11.32)[17]	nuc ish (DXZ1x1)[168]//(DXZ1x2)[14]//(DXZ1,DYZ3)x1[30]//(DXZ1x2,DYZ3x1)[7]//(DXZ1x1,DYZ3x2)[76]//(DXZ1x2,DYZ3x2)[4]//(DXZ1x1,DYZ3x3)[1]	1

15.	mos 45,X[2]/46,X,idic(Y)(p11.32)[28]	nuc ish(DXZ1x1)[200]	1

16.	mos 45,X[4]/46,X,del(Y)(q10)[34]/46,X,del(Y)(p10)[1]/47,X,chrb(Y)(q10)[1]	nuc ish(DXZ1x1)[72]//(DXZ1,DYZ3)x1[228]	1

17.	mos 45,X,chtb(3)(p14.2)[1]/47,XY,+2[1]/47,XY,+21[1]/45,XY,-18[1]/45,XY,-20[1]/46,XY,chtb(3)(p14.2)[4]/46,XY,chtb(14)(q22)[1]/46,XY[30]	nuc ish(DXZ1,DYZ3)x1,(D18Z1x2)[282]//(DXZ1,DYZ3)x1,(D18Z1x1)[18] and nuc ish(DXZ1,DYZ3)x1,(RB1x2)[294]//DXZ1,DYZ3)x1,(RB1x1)[6]	1

18.	mos 45,X[13]/47,XXY[27]	nuc ish(DXZ1x1)[75]//(DXZ1,DYZ3)x1[66]//(DXZ1,DYZ3x2)[159]*	1

FISH: Fluorescence In Situ Hybridization

Most of the cases were referred at the age of puberty (8-13 years old) or beyond (14-18 years old), 31 and 21 cases, respectively (Table 3), and interestingly there were 14 cases were sent in adulthood (> 18 years old). Even pure 45,X cases were mostly referred at the age of puberty or beyond (12, 14, and 4 cases referred at the age of 8-13 years, 14-17 years, and > 18 years old, respectively), and patients with mosaic 45,X/46,XY or Y segments were sent at younger age (8 cases out of 21 in infancy).

**Table 3:** Distribution of age at referral and referral reasons for chromosome analysis

Age	45,X	mos 45,X/46,XX or with part of X	mos 45,X/46,XY or with part of Y	Total
0-11 months	5	3	8	16

1-7 years	2	4	3	9

8-13 years	12	14	5	31

14-17 years	14	5	2	21

≥ 18 years	4	8	2	14

Not available	2	1	1	4

**Referral reasons**				

Short stature	11	13	2	26

Primary amenorrhoe	8	4	2	14

Dysmorphic features (other Turner stigmata)	9	1	2	12

Delayed puberty	2	3	1	6

History of trisomy in previous pregnancy/child	0	2	0	2

Genitalia ambiguity	0	1	8	9

Other	0	2	0	2

Not available	16	11	11	38

The frequent reasons to refer patients for chromosomal analysis in 45,X cases were short stature, primary amenorrhea, presence of other Turner syndrome stigmata, and ambiguous genitalia (Table 3). For mos 45,X/46,XX cases, they were mostly referred because of short stature (13 cases), and for mos 45,X/46,XY or mosaic with other Y segments, because of genitalia ambiguity (8 cases).

## Discussion

Monosomy × or Turner syndrome is one of the most common chromosomal abnormalities, which occurs in around 1:3000 live birth in girls. In around 50% of patients with TS, the karyotypes anomaly is monosomy X, but other chromosomal anomalies have been detected, including mosaicism, Xp or Xq deletion, and isochromosomes of the long arm of × chromosome [1-3]. In our series, 41.1% of cases showed non-mosaic monosomy × chromosomal analysis result (Table 1). This result lead the 45,X as the most frequent chromosome aberration found. Similarly, other report from Serbia revealed non mosaicism 45,X in 48.4% of 31 patients with Turner syndrome stigmata [7]. The next most frequent sex chromosomal anomalies found in this report was mosaicism containing 45,X/46,X,i(Xq) aberration, which accounted in 14/95 cases (14.7%), while other report by Sybert and McCauley [3] found the frequency of 7% and 8% for 46,X,i(Xq) and mosaic 45,X/46,X,i(Xq), respectively. According to Djordjevic's report [7] aberration i(Xq) was present in 7/31 (22.6%), which included 46,X,i(Xq) in 5/31(16.1%) patients. These findings deviated from our results, which did not detect any 46,X,i(Xq), and we assumed that the 45,X cell populations were likely to be generated by mitotic loss of the iXq chromosome.

With standard chromosome analysis, which based on cell cultivation for metaphase spread preparations, followed by painting with specific stains for having specific banding of chromosomes, the conventional cytogenetic techniques allows the visualization of cellular karyotype, but this technique do not provide detection of genomic variations involving DNA sequences smaller than 3-5 Mb. Additionally, cell lines carrying chromosome aberrations most likely do not survive during cell cultivation process, which may lead to improper results. Molecular cytogenetic techniques are expected to overcome the conventional cytogenetic shortcomings and FISH is one of the most applied molecular cytogenetic techniques. Interphase FISH is a valuable set of techniques for uncovering intercellular genomic variations in non-cultivated cells. Furthermore, another advantage of FISH is the ability to provide cytogenetic analysis for large cell populations. There are two FISH-based approaches in order to get better resolution in examining mosaicism, which are quantitative FISH (QFISH) and interphase chromosome-specific multicolor banding (ICS-MCB). The molecular cytogenetic techniques are highly efficient for diagnosis of numerous diseases associated with brain dysfunction [8,9].

Recently, the significant achievement in the field of molecular cytogenetic has brought evidences that demonstrated a higher incidence of chromosomal mosaicism in diseased individuals, i.e. brain diseases [8], and additionally, chromosomal mosaicism is not just a casual finding during cytogenetic analysis, but a more significant biological phenomenon than previously recognized and its roles in genetic diversity, human diseases, abnormal prenatal development are still to be elucidated [10]. In our study we did not perform further analysis for non mosaic monosomy × detected by 20 metaphase cells conventional cytogenetic analysis, based on the opinion that extensive searching for 46,XX cells in 45,X karyotype individuals is not necessary, since the detection of a normal cell lineages in fewer than 5 percent of cells does not change the prognosis and management [3]. Interestingly, in our study among cases underwent both karyotyping and FISH there were 7 cases had normal 46,XX or 46,XY karyotypes, but had mosaic or additional cell lineages with monosomy × detected using FISH and the level of mosaicism was between 3% to 10%. This can lead to misdiagnosis and is a challenging situation, which may be encountered by clinician and should lead them to be more cautious in facing cases with clinical suspicion of TS, but otherwise normal results of karyotypes. It is still a major problem to interpret mosaicism, especially in cases with low-level mosaicism. Further evaluation to detect hidden 45,X cell line using sex chromosome FISH or other molecular cytogenetic methods should be considered. As proposed by Vorsanova et al. [11] FISH and CGH (comparative genomic hybridization) might be necessary in chromosomal disorders in order to provide higher detection rate of somatic chromosomal mosaicism. If the diagnosis of TS is suspected clinically but the result of routine testing is normal, it is indicated to increase the number of cells counted to 100 and to perform a skin biopsy for fibroblast karyotyping to rule out mosaicism for an abnormal cell lineage [3]. Other group [12] recommended in such cases cytogenetic study of a second tissue (e.g. skin biopsy for cell culture or buccal smear for FISH).

Most of our cases (58.9%) have mosaic 45,X cytogenetic analysis results, which assumed to contribute to the phenotypes diversity found. Many authors believed that mosaicism formations occur during fetal life. Spontaneous somatic chromosomal variations presenting as low level mosaicism can be detected in all somatic cell populations. However, the low-level mosaicism is frequently overlooked, because of unapparent phenotypic effects. On the other hand Yurov et al [13], showed that human developing brain has mosaic nature, being composed of euploid and aneuploid cells, and determined the average aneuploidy frequency as 1.25-1.45% per chromosome with the overall percentage of aneuploidy tending to approach to 30-35% and suggested there is an expected link between developmental chromosomal instability, intercellular/intertissular genome diversity and human brain diseases. Similarly a systematic review and meta analysis [14] on the chromosomal constitution of human preimplantation embryos reported 73% of embryos were mosaic, of which diploid-aneuploid mosaic was the most prevalent type of mosaicism (59%) detected. In view of relationship between the diversity in cytogenetic results and the phenotypes, surely our study samples still need to be explored further using combination of molecular cytogenetic methods. In addition with the use of molecular cytogenetics the possible relationship between genomic variations and monosomy × phenotypes will be clarified.

The use of fluorescence in situ hybridization may increase the prevalence of sex chromosomal mosaicism detection in non-mosaic 45,X cases [9,11]. By combining karyotyping and FISH methods, we obtained 22 out of 31 cases (70.9%) had different results, including detection of additional XYY cell line by FISH in two mosaic X/XY and one X/XXY karyotyped cases (asterisks in Table 2), while through karyotyping technique structural chromosomal anomalies, such as delXp, iXq, delYp, rX, rY, etc, which found to be a normal signal of × or Y by FISH, can be detected (Table 2). These data convince us to perform both karyotyping and FISH in 45,X cases routinely, because different constitutions of sex chromosome aberrations result in variable clinical characteristics, for instance, individuals with iXq have increased risk of autoimmunity, particularly thyroiditis and inflammatory bowel disease, and deafness [15], and in group 45,X/46,XX the sex chromosome mosaicism is responsible for clinical changes from 6% aneuploidy, corresponding to the main phenotypical features of TS [16]. These phenomena will lead to different management approaches.

With conventional cytogenetic analysis, the possibility of mosaicism cannot be excluded without large numbers of mitoses being examined. For karyotyping, we used to analyze 20 metaphase cells, but learning from this experience, to rule out sex chromosome mosaicism, we should analyze more, at least 30 metaphase cells using conventional cytogenetic as recommended by The American College of Medical Genetics [12]. Furthermore, invitro cell selection may affect the percentage of cells in a given karyotype. In this report we found 33/39 of these 45,X cases were detected by karyotyping only, which did not absolutely exluded the possibility of mosaicism. In this case, FISH as an adjunct assay to conventional cytogenetic technique plays important role in detecting low level mosaicism.

The presence of Y chromosome fragments in patients with TS is known to increase the risk of gonadoblastoma [1,6,17]. In this report, we found 22.1% of 45,X cases had Y or part of Y chromosome, which mostly referred at younger age because of genitalia ambiguity (Table 3). This percentage was higher than other study reported by other groups [6,17]. This discrepancy may be explained by disparity in the study methods, we included all the cases with 45,X cytogenetic result disregard of the phenotypes, while other reports analyzed only TS females.

**Figure 1:**
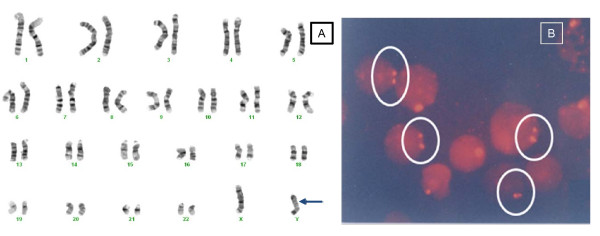
**Karyotype and FISH with Y chromosome specific probe of a 'girl' with isodicentric Y chromosome**. 'She' (aged 20 months old) presented with ambiguous genitalia (enlarged phallus, no palpable testis). 'She' had functioning test revealed by HCG test. The results of cytogenetic analysis showed mos 45,X[1]/46,X,idic(Y)(p11.32)[17] karyotype and nuc ish (DXZ1x1)[168]//(DXZ1x2)[14]//(DXZ1,DYZ3)x1[30]//(DXZ1x2,DYZ3x1)[7]//(DXZ1x1,DYZ3x2)[76]//(DXZ1x2,DYZ3x2)[4]//(DXZ1x1,DYZ3x3)[1]. Panel A shows the 46,X,idic(Y)(p11.32) karyotype and Panel B indicates the Y chromosome signals, which always appears side by side in some cells.

Many studies reported that these Y chromosome aberrations, especially isodicentric Y chromosome can produce chromosome mosaicism, due to their instability during cell division, and consequently alternate cell lines, generally including 45,X (95% cases) may be generated [18,19]. We found 9/95 cases (Table 1 and Table 2) whose karyotypes consisted of Y chromosome aberrations, including isodicentric Y chromosome in 2/95 cases. The cytogenetic results of a 20 month-'girl' with isodicentric Y chromosome showed in Figure 1. Detailed analysis with a series of high resolution molecular cytogenetic techniques may revealed possible mechanisms of chromosomal aberrations, as well as explained genotype phenotype relationships [18,19].

Despite different level of mosaicism found using karyotyping and FISH, in our report three cases (asterisks in Table 2) were found to have additional cell population (XYY) detected by FISH with 8% and 53% level of mosaicism, which were revealed mos 45,X/46,XY and 45,X/47,XXY by karyotyping. Bianco B et al. [20] reported analysis of different tissues of 45,X karyotype patients, revealed seven (35%) out of 20 patients presented hidden chromosome Y mosaicism. Four of these patients underwent prophylactic gonadectomy, and bilateral gonadoblastoma was found in one of them. Therefore, careful analysis of Y chromosome fragments is necessary.

Different from other studies, we found only 16/95 (16.8%) of 45, × cases detected in infancy and most of the cases were referred for chromosomal evaluation at the age of puberty and beyond (Table 3). Interestingly, in our series 14 cases, of which 8 were mos 45, X/46,XX or other forms of mos for 45, × with × segments, were sent in adulthood. Even pure 45, × cases were sent late. The resulting phenotype of 45, × individuals varies according to the underlying chromosomal constitution, but short stature is considered to be the most prominent cardinal features of this disorder. However, significant ascertainment bias exists. A case of non-mosaic 45, × girl with tall stature (170 cm) diagnosed at 18 years old, was reported recently. The only apparent Turner stigmata she presented was gonadal dysgenesis, which caused the diagnosis delay [21].

Despite the possibility of hidden mosaicism in pure 45,X cases, that might lead to milder phenotypes and therefore were missed, looking at our data, it seems that for most of the cases, the awareness of clinicians or the parents rose after significant clinical signs, such as short stature, primary amenorrhea, lack of secondary pubertal signs, appeared (Table 3). While cases referred at young age mostly due to genitalia ambiguity, which is an obvious clinical sign and a social emergency for the family, and therefore needs prompt diagnosis and management. In terms of TS management, especially for growth hormone treatment, these data had clinical importance, because growth response is negatively correlated with age at the start of therapy [22,23] and similar to girls with TS, X/XY children with short stature also benefit from growth hormone treatment, especially when it initiates early [23]. Combination of karyotyping and FISH is expected to support the clinician in establishing the diagnosis of this condition early by providing details on the sex chromosome constitution.

In conclusion, using combination of conventional cytogenetic and FISH, we detected a wide variation of sex chromosome aberrations, including detection of low level of mosaicism and Y-chromosome fragments. Result discrepancies may be produced using both techniques and in order to obtain a more details of sex chromosome constitution of individuals with 45, × cell line, both FISH and karyotyping should be carried out simultaneously. Further studies using combination of molecular cytogenetic methods, including MCB and CGH may provide higher detection rate of mosaicism and the possible explanation of the wide variations of TS phenotypic corresponding to its diversity in somatic chromosomal constitutions, as well as the mechanism of sex chromosome aberrations.

## Methods

### Cases

Subjects of this study were drawn from all cases referred to our clinic between year 2005 until 2009 and whose cytogenetic results contained monosomy × cell populations. Cytogenetic results and data of the subjects were analyzed. We look for the variations of sex chromosomes abnormalities in individuals with monosomy × cell line and the results differences using conventional cytogenetic technique and Fluorescence in situ hybridization (FISH).

### Cytogenetics

Metaphase chromosomes from blood lymphocytes were prepared according to standard procedures [24]. Chromosome analyses were performed applying GTG Banding [25] at a 500 band level according ISCN 2009 [26] with the average number of 20 metaphase cells. Up to 40 metaphase cells were analyzed, when mosaicism found in routine karyotyping.

### Fluorescence in situ hybridization (FISH)

FISH was carried out as standard procedures according to probes manufacturer manual, using CEPX(DXZ1)/Y(DYZ3) probes (*Vysis*, USA) for up to 300 interphase cells.

## Competing interests

The authors declare that they have no competing interests.

## Authors' contributions

NSM counseled the patients, collected and analyzed the data, drafted, revised, and finalized the manuscript. HWA carried out the karyotyping. LPS participated in data collection, carried out the karyotyping. DDA carried out the FISH analysis. CP performed the karyotyping and FISH analysis, involved in manuscript revision. HK performed the karyotyping, and FISH analysis, helped to draft the manuscript, ABP counseled the patients, involved in analyzing the data, and revising the manuscript. AH supervised the cytogenetic results, involved in revising the manuscript, and gave the final approval of the manuscript. All authors read and approved the final manuscript.
